# Association between ischemic stroke and migraine in elderly Chinese: a case–control study

**DOI:** 10.1186/1471-2318-13-126

**Published:** 2013-11-19

**Authors:** Haijun Li, Ying Yu

**Affiliations:** 1 Department of Neurology, Municipal Hospital of Taizhou, Jiaojiang, Zhejiang 318000, China

**Keywords:** Migraine, Acute cerebral infarction, Elderly Chinese, Aura

## Abstract

**Background:**

Recent observations suggest that migraine and cerebrovascular disease are comorbid conditions. However, the association of migraine with cerebrovascular disease in the population of elderly Chinese has not been established. This prospective case–control study aimed to investigate the prevalence and lesion characteristics of migraine in elderly Chinese patients with acute cerebral infarction (ACI).

**Methods:**

A total of 968 ACI patients aged 55–70 years and 1024 sex- and age-matched control subjects were recruited between January, 2003 and July, 2009. Migraine was determined based on the International Headache Society’s Classification of Headache Disorders, together with past medical records and admission examination results, following an initial questionnaire screening at the first hospital visit. Prevalence rates of overall migraine, migraine with aura and migraine without aura in both ACI patients and control subjects, the stroke subtypes classified according to the Chinese Ischemic Stroke Subclassification (CISS) system and brain locations of the ischemic lesions in ACI patients were analyzed.

**Results:**

The overall prevalence rate of migraine was 17.15% (166/968) in patients with ACI but only 3.9% (40/1024) in control subjects (P < 0.01). In both subject groups, over 80% of migraine cases were migraine without aura. In the 166 ACI patients with comorbid migraine, large artery atherosclerosis was the most frequent subtype of ischemic lesion (65.06%), followed by cardiogenic stroke (23.50%), and all other lesion subtypes were each less than 10%. Ischemic infarctions were located predominantly in the anterior circulation in the brain in both ACI patients with and without migraine.

**Conclusions:**

The prevalence rate of migraine is significantly higher in ACI patients than non-ACI subjects in the population of elderly Chinese. Migraine without aura is the major form of migraine in both ACI patients and non-ACI subjects. In ACI patients, regardless of migraine, infarction lesions were predominantly located in the anterior cerebral circulation.

## Background

Migraine is a common chronic neurological disorder that is characterized by moderate to severe headache pain and often accompanied by gastrointestinal and autonomic nervous system disturbances [1,2]. According to the International Classification of Headache Disorders established by the International Headache Society [3], migraine with aura and migraine without aura are the two most common types of migraine; less common types include abdominal migraine, basilar artery migraine, carotidynia, headache-free migraine (aura without migraine), ophthalmoplegic migraine/ocular migraine, and status migrainosus.

Although the eitopathogenesis of migraine remains incompletely understood, it is now increasingly recognized that migraine is more complex than a sole vascular disease as previously thought [4-6]. Recent neuroimaging studies suggests that migraine and cerebrovascular disease are comorbid conditions. In a case–control study involving 314 stroke patients and 314 control subjects aged 16–44 years, Camerlingo et al. demonstrated that migraine with aura was associated with ischemic stroke in young women but not men [7]. In the CAMERA study, a community-based study conducted in the Netherlands involving 295 migraine patients and 140 controls aged 30–60 years, a cross-sectional association was demonstrated between migraine and silent infarct-like lesions in the posterior circulation area of the brain [8]. To date, the majority of the studies on the association between migraine and ischemic infarction/stroke have been performed on people at the age of 60 years or younger; little is known regarding the association of ischemic infarction/stroke with migraine in the elderly, particularly in the elderly Chinese. This study aimed to compare the prevalence of migraine between elderly Chinese patients with acute cerebral infarction (ACI) and the age- and gender-matched control subjects without ACI. Cerebral locations of infarction lesions in ACI patients were also assessed.

## Methods

### Ethical considerations

The study was reviewed and approved by the Institutional Ethics Committee of the City Hospital of Taizhou and conducted in compliance with the Helsinki Declaration. Written informed consent was obtained from all subjects.

### Study design and subjects

A prospective case–control study was designed. A total of 968 patients with cerebrovascular disease who visited the Department of Neurology of the City Hospital of Taizhou in Zhejiang, China between January 2003 and July 2009 were consecutively recruited. The inclusion criteria were acute cerebral infarction (ACI) and age between 55 and 70 years. ACI was diagnosed by attending neurologists according to the criteria adopted at the Fourth Chinese National Stroke Conference in 1995 [9] and confirmed by cranial CT or MRI scan. Based on the MRI results, ACI patients were classified into five subtypes: 1) large artery atherosclerosis (LAA), 2) cardiogenic stroke (CS), 3) penetrating artery disease (PAD), 4) other etiology (OE), and 5) undetermined etiology (UE), according to a new etiology- and mechanism-based Chinese Ischemic Stroke Subclassification (CISS) system [10]. Patients with brain tumors, aneurysms, and meningitis were excluded. A total of 1024 sex- and age-matched patients who were hospitalized with infectious diseases during the same time period were included as controls. Both ACI patients and control subjects received in-hospital treatments specific for their conditions.

### Assessment of migraine

Prevalence of migraine was the primary measurement of this case–control study. An initial migraine screening was performed on all subjects through a standardized interview at the patients’ first hospital visits. Those who reported migraine attacks were further assessed according to the International Classification of Headache Disorders established by the International Headache Society (IHS) [3], together with a comprehensive review of the past medical records and admission examination results. Types of migraine (i.e. migraine with and without aura, formerly known as “classic” and “common” respectively) were determined by the IHS criteria. The proportions of stroke subtypes and brain locations (i.e. anterior and posterior circulations) of ischemic infarction in ACI patients with and without migraine respectively were analyzed. Information on the course of migraine, times of attack, frequency of seizure, treatments, the family history of acute cerebral infarction and stroke, smoking status was collected. Blood pressure, blood levels of cholesterol, homocysteine, and glucose were all measured immediately after hospital admission.

### Statistical analysis

Data are presented as mean and standard deviation. They were analyzed by chi square test. Differences were considered significant if P < 0.05. The statistical analysis software SPSS (Chicago, IL, USA) was used.

## Results

### Basic demographics and major stroke risk factors

The average age was 64.2 ± 10.1 (60–68) years in patients with ACI and 58.1 ± 11.4 (55–63) years in the control subjects. The difference failed to reach a statistically significant level (P > 0.05). In the 968 ACI patients, there were 517 men and 451 women. Of the 1024 control subjects, 514 were men and 510 were women. The male/female ratio was not significantly different between ACI patients and control subjects (P > 0.05). In addition, the percentage of individuals with hypertension, diabetes, high hyperhomocysteinemia, high cholesterol, carotid artery plaque, unhealthy behaviors (i.e. smoking 20 cigarettes/day and drinking half a catty/day) or other risk factors for stroke was not significantly different between the two groups of subjects (P > 0.05).

### Prevalence of migraine

Summarized in Table 1 are the results of migraine assessment in ACI patients and control subjects. Migraine was diagnosed in 166 of the ACI patients and in 40 of the control subjects; the prevalence rate of migraine was significantly higher in the ACI group (17.15%) than in the control group (3.90%) (P < 0.01). The vast majority of migraine cases were migraine without aura in both groups of subjects.

**Table 1:** Prevalence and duration of migraine in ACI patients and control subjects

**Subjects**	**Prevalence of migraine**	**Without aura**	**With aura**	**Duration (yrs)**
ACI	17.15% (166/968)**	81.9% (136/166)	18.9% (30/166)	18.40*
Control	3.90% (40/1024)	85% (34/40)	15% (6/40)	14.70

Double asterisks (**) indicate a significant difference from the control group at P < 0.01 and a single asterisk (*) indicates a significant difference from the control group at P < 0.05.

### Proportions of different stroke subtypes and cerebral locations of ischemic infarction

Shown in Table 2 are results on the proportions of the CISS stroke subtypes and ischemic infarction locations in anterior and posterior circulations in elderly ACI patients with (n = 166) and without (n = 802) migraine. Regardless of migraine, large artery atherosclerosis (LAA) was the most frequent subtype of ischemic lesion, followed cardiogenic stroke (CS), penetrating artery disease (PAD), other etiology (OE), and undetermined etiology (UE). The proportion of the LAA subtype was significantly lower (P < 0.05) but the proportion of the CS subtype was significantly higher (P < 0.05) in ACI patients with migraine than in those without migraine, while the differences in the other stroke subtypes failed to reach a statistically significant level (P > 0.05). In ACI patients both with and without migraine, ischemic infarctions were detected in the anterior circulation of the brain in over two thirds of the cases, consistently in all stroke subtypes.

**Table 2:** Proportions of different stroke subtypes classified according to the Chinese Ischemic Stroke Subclassification (CISS) criteria in 968 acute cerebral infarction (ACI) patients with and without migraine respectively and cerebral locations of ischemic infarction in different stroke subtypes

	**ACI patients with migraine (n = 166)**			**ACI patients without migraine (n = 802)**		
**Subtype**	**Overall frequency**	**Anterior circulation**	**Posterior circulation**	**Overall frequency**	**Anterior circulation**	**Posterior circulation**
**LAA**	65.06%*	63.89% (69/108)	36.11% (39/108)	86.16%	65.12% (450/691)	34.88% (241/691)
**CS**	23.50%*	66.67% (26/39)	33.33% (13/39)	8.35%	73.13% (49/67)	26.86% (18/67)
**PAD**	6.02%	80.00% (8/10)	20.00% (2/10)	2.62%	71.43% (15/21)	28.57 (6/21)
**OE**	3.61%	66.67% (4/6)	33.33% (2/6)	1.87%	66.67% (10/15)	33.33% (5/15)
**UE**	1.81%	100% (3/3)*	0% (0/0)	1.00%	87.50% (7/8)	12.50% (1/8)

*LAA* Patients with large artery atherosclerosis, *CS* Cardiogenic stroke, *PAD* Penetrating artery disease, *OE* Other etiology, and *UE* Undetermined etiology. A single asterisk (*) denotes that the difference in the same variable for the same stroke subtype was significant between ACI patients with migraine and ACI patients without migraine at α = 0.05.

## Discussion

Migraine, together with tension-type headache, cluster headache and medication overuse headache, is among the most common primary headache disorders. Globally, the overall prevalence rate of migraine ranges from 0.9% to 5.1% in the general population [11]. In the present study, migraine was diagnosed in 40 out of the 1024 elderly control subjects, with a prevalence rate of 3.9% that was in the range of the global estimate. In contrast to the control subjects, patients with ACI had a much higher prevalence rate of migraine (17.15%). It has been well documented that the prevalence rate of migraine varies with age and gender [12]. Although several previous studies in the literature assessed the association between migraine and cerebral infarction, those studies largely involved young populations [7,8]. In the present study, we have for the first time shown that elderly Chinese patients with cerebrovascular disease are at higher risk of developing migraine.

As previously shown in young populations, the association between migraine and cerebral infarction is independent of risk factors for stroke [7]. In the present study, we also collected and analyzed the data on major stroke risk factors, but no significant differences in the prevalence of hypertension, diabetes, high hyperhomocysteinemia, high cholesterol, carotid artery plaque as well as in smoking and drinking behaviors were observed between the ACI patients and the control subjects. This observation suggests that the association between migraine and cerebral infarction might be also independent of stroke risk factors in the elderly.

In the present study, we also assessed the proportions of different subtypes of ischemic infarction as well as lesion locations in the brain for each of the subtypes in the ACI patients. LAA was the dominant subtype of cerebral infarction, followed by CS, in the ACI patients, regardless of migraine. Lesions were detected more frequently in the anterior circulation than the posterior circulation of the brain. These novel observations suggest that artery atherosclerosis in the anterior circulation of the brain is the major type of ischemic lesion in the elderly Chinese patients with ACI.

To date, the mechanism underlying migraine and cerebral infarction remains inconclusive. Some observational studies have shown that migraine with aura is often accompanied by cortical spreading depression (Cortical Spreading Depression, CSD). Intracranial vasoconstriction and cerebral ischemia accompanied by CSD may impair metabolic function of the brain and cause local redistribution of intracellular and extracellular ions, thus leading to brain cell swelling and neuron injury.

Posterior cerebral artery is the densest part of cerebral arterial circle, making the occipital lobe more prone to infarction [13]. It has been documented that 81% of all infarct-like lesions in migraine patients with aura are localized in the posterior cerebrum but this value is 47% in migraine patients without aura [14].

It has been suggest that the relationship between migraine and cerebral infarction is bidirectional. However, the present study only attempted to address the question whether cerebrovascular disease is associated with increased risk of developing migraine in elderly Chinese. As a result, our observations allow no conclusions on whether migraine is associated with increased risk of ischemic cerebral infarction/stroke.

Another limitation of the present study was the use of patients with infectious diseases rather than healthy volunteers as control subjects. Although these control subjects did not have any neurovascular lesions, we could not totally rule out the possible impact of infections on the development of migraine, which might underestimate the difference in the prevalence of migraine between two groups.

## Conclusions

The present study has for the first time shown that the prevalence rate of migraine is significantly higher in elderly Chinese patients with acute cerebral infarction. Given that migraine is a disorder underdiagnosed and undertreated and that the Chinese population is rapidly aging with increased risk of developing cerebrovascular disease, a better understanding of the association of migraine with ischemic cerebral infarction would be helpful in the development of potential preventive and therapeutic strategies for migraine in elderly Chinese.

## Competing interests

The authors declare that they have no competing interests.

## Authors’ contributions

HJL defined the research theme. HJL and YL designed methods and experiments, carried out the laboratory experiments, analyzed the data. Both authors read and approved the final manuscript.

## Pre-publication history

The pre-publication history for this paper can be accessed here:

http://www.biomedcentral.com/1471-2318/13/126/prepub
